# Supplementary material for: Kinetics of change in lipid hydroperoxides and conjugated dienes as affected by temperature: Shelf-life estimation of vegetable oils
Source: Food Chem X. 2026 Oct 10;39:104586. doi: 10.1016/j.fochx.2026.104586 (PMC13675803; doi:10.1016/j.fochx.2026.104586)
Supplement: Supplementary file 1 [file mmc1.docx]

**Kinetics of change in lipid hydroperoxides and conjugated dienes as affected by temperature: Shelf-life estimation of vegetable oils**

Negin Rahimzadeh, Zahra Hoseinipoya, and Reza Farhoosh^*^

Department of Food Science and Technology, Faculty of Agriculture, Ferdowsi University of Mashhad, P.O. Box 91775-1163, Mashhad, Iran.

^*^ Correspondence: [rfarhoosh@um.ac.ir](mailto:rfarhoosh@um.ac.ir)

**Abstract**

The impact of temperature (45 – 85°C) on the kinetics of simultaneous change in the concentrations of lipid hydroperoxides (LOOH) and conjugated dienes (CD) during the initiation and propagation peroxidation phases of olive, corn, sesame, and canola oils was investigated. The LOOH- and CD-based values of the same kinetic and thermodynamic parameters in each oil provided a number of valuable mechanistic information regarding the non-conjugated/conjugated diene hydroperoxides and conjugated diene hydroperoxides/non-hydroperoxides, respectively. The results demonstrated that both the LOOH- and CD-based kinetics of lipid peroxidation at low temperatures (< 60°C) or even at relatively high temperatures (up to ~90°C) can practically be used to estimate model-predicted shelf-lives under the specified experimental conditions.

**Keywords**: Arrhenius and Eyring equations, induction period, lipid peroxidation, rate constant, temperature coefficient.

**1. Introduction**

For decades, the free radical chain mechanism of lipid peroxidation has been explaining how lipid hydroperoxides (LOOH) as the fundamental primary oxidation products are generated through abstracting hydrogen atoms at *mono*- or *bis*-allylic carbons of unsaturated lipids (Schaich, 2005). LOOH in oxidized vegetable oils include non-conjugated (nCDOOH) and conjugated (CDOOH) diene hydroperoxides arising from mono- (MUFA) and polyunsaturated (PUFA), respectively, fatty acids (Frankel, 2012a). The total amount of LOOH (nCDOOH + CDOOH) is reliably measured by the spectrophotometric thiocyanate technique (Shantha & Decker, 1994). UV absorbance at 234 nm can also be used to determine the concentration of CDOOH alone (Pegg, 2001). As the reaction progresses towards advanced levels, LOOH decompose more significantly into a wide variety of non-hydroperoxide derivatives that are no longer detectable by the thiocyanate technique. However, the decomposed CDOOH may provide conjugated diene non-hydroperoxides (CDnOOH) that still absorb at 234 nm. Accordingly, the spectrophotometric method yields actually the total amount of CDOOH and CDnOOH, symbolized as CD (Jaldani, Sarfaraz Khabbaz, Jooyandeh, & Farhoosh, 2024).

The same classical kinetic patterns have been observed for the changes in LOOH and CD concentrations over peroxidation of edible fats and oils, so that they increase linearly during an initiation phase (induction period, IP) and reach the critical concentrations [LOOH]_IP_ and [CD]_IP_ in the end (Farhoosh, 2020) (Fig. 1). Lipid peroxidation in the phase is characterized by a pseudo-zero order rate law (Kamal-Eldin & Yanishlieva, 2005) with the rate constant *k*_IP_. In the propagation phase following the reaction, [LOOH]_IP_ and [CD]_IP_ increase rapidly to the ultimate concentrations [LOOH]_max_ and [CD]_max_, respectively. The phase has been described well by a sigmoidal kinetic model providing two helpful composite (*k*_c_ of pseudo-first kinetic order) and decomposition (*k*_d_ of pseudo-second kinetic order) rate constants (Farhoosh, 2018; Farhoosh, 2020). Further kinetic changes in [LOOH] and [CD] occur during a termination phase which lacks any practical value in applied research.

The kinetics of simultaneous changes in [LOOH] and [CD], which stand for some different types of peroxidation species, will provide invaluable information regarding the response of lipid systems to any changes in environmental conditions such as temperature (Jaldani, et al., 2024). Temperature has been shown to exponentially affect the kinetics of LOOH accumulation (Frankel, 2012b). The kinetic parameters representing the initiation and propagation phases of LOOH accumulation are temperature-dependent. The temperature coefficient *T*_C_ shows how quickly the kinetic parameters vary with temperature. Besides, the well-known Arrhenius and Eyring equations have been used to investigate dependency of the LOOH-based rate constants *k*_IP_, *k*_c_, and *k*_d_ on temperature (Farhoosh, 2021). However, literature review provided no data on the changes in CD-based kinetic parameters as a function of temperature.

The temperature dependence of the LOOH- and/or CD-based kinetic parameters can potentially be applied to predict oxidative stability of lipid systems under various heat processing, storage, and distribution conditions. Shelf-life of bulk phase oils has frequently been estimated by plotting the logarithm of IP versus some accelerated temperatures and then extrapolating to ambient temperatures (Frankel, 2012a). Fundamentally, shelf-life estimations require investigating the regression of peroxidation rate constants on accelerated temperatures according to the kinetic Arrhenius model as well as possessing an appropriate cut-off value for rejection (Farhoosh, 2022). Our recent studies revealed that some critical concentrations of hydroperoxide species located in the propagation phase of lipid peroxidation may be used as the cut-off values for rejection purposes (Moosavi & Farhoosh, 2023; Sarfaraz Khabbaz, Jooyandeh, Jaldani, & Farhoosh, 2024).

The range of temperatures used in the studies related to the oxidative stability of lipid systems has always been of crucial importance. The mechanism of lipid peroxidation is postulated to be significantly different at temperatures higher than 60°C. This has been attributed to more dependency of the reaction on oxygen concentration at higher temperatures (Gomez-Alonso, Salvador, & Fregapane, 2004). Frankel (2012b) reported that the solubility of oxygen in oil or water decreases by ~25% for every 10°C increase. The temperature dependence of the mechanism of the reaction might be eliminated by performing oxidative stability tests in a kinetic regime of oxidation (Farhoosh, 2022). In such a condition, which is created by blowing oxygen or air through oil, or by performing the reaction in thin layers (*<*1 mm), peroxidation would be expected to be independent of oxygen concentration and provide kinetic data of higher reproducibility (Yanishlieva & Marinova, 2003). In addition, the level of temperature used for oxidative stability tests depends greatly on the fatty acid composition of the oil experimented. In general, lower temperatures in the range of 40–60°C for vegetable oils are suggested to study the oxidative stability of more polyunsaturated oils. According to Gomez-Alonso et al. (2004), the results of oxidative stability tests in this temperature range correlate satisfactorily with those of storage at room temperature or potential shelf-lives.

The present study aimed to comparatively investigate the impact of temperature on the kinetics of simultaneous change in [LOOH] and [CD] during lipid peroxidation. The temperatures studied ranged from those below to above 60°C (45–85°C) in a kinetic regime of oxidation. Some selected vegetable oils of different fatty acid composition, representing the oleic (olive oil), oleic-linoleic (corn and sesame oils), and linolenic (canola oil) groups of vegetable oils, were exposed to the oxidative stability tests. In the end, the kinetic parameters obtained were employed to estimate shelf-lives.

**2. Materials and methods**

*2.1. Materials*

Commercial olive, corn, sesame, and canola oils were purchased from local Markets and stored at –18°C until analysis. Peroxide (PV, according to Shantha & Decker, 1994) and acid (AV, according to AOCS, 2017) values of the oils were less than 3.0 mEq kg^–1^ and 0.4 mg g^–1^, respectively, indicating their appropriate initial quality. The fatty acid composition (according to Farhoosh, Niazmand, Rezaei, & Sarabi, 2008) of the olive, corn, sesame, and canola oils mainly consisted of palmitic (13.6, 9.97, 9.35, and 4.85%, respectively), stearic (2.28, 2.36, 4.73, and 2.15%, respectively), oleic (64.0, 31.2, 38.3, and 62.5%, respectively), linoleic (16.7, 53.9, 45.8, and 20.1%, respectively), and linolenic (0.70, 0.92, 0.41, and 7.63%, respectively) acids. The olive, corn, sesame, and canola oils contained 385, 541, 285, and 617 ppm tocopherols (according to Wong, Timms, & Goh, 1988) and 267, 109, 126, and 271 ppm phenolic compounds (according to Capannesi, Palchetti, Mascini, & Parenti, 2000), respectively. As taken from the labels of the oil bottles, the corn and canola oils also contained some noticeable amounts of the added synthetic antioxidant TBHQ (75 ppm) and the synergist citric acid (100 and 50 ppm, respectively). All the chemicals and solvents used in the study were of analytical reagent grade and purchased from Merck (Darmstadt, Germany) and Sigma-Aldrich (St. Louis, MO. USA).

*2.2. Peroxidation*

The oils (13.5 g in 1-mm layers) in Petri dishes (15 cm in diameter) were oxidized in a kinetic regime of oxidation at 45, 55, 65, 75, and 85°C (± 3°C, dry ventilated oven, A&D Co.) (Yanishlieva & Marinova, 2003). [LOOH] (mM, 0.504 × PV) and [CD] (mM) were measured over oxidation. To measure PV (Shantha & Decker, 1994), the oils (0.01–0.30 g, depending on the extent of peroxidation) were vortexed with chloroform–methanol (7:3 v/v, 9.8 mL) for 2–4 s. After adding a solution of ammonium thiocyanate (30% w/v, 50 µL), the solutions were vortexed again (2–4 s). Fifty microliter of a clear solution of FeCl_2_ ([0.4 g barium chloride dihydrate dissolved in 50 mL H_2_O]+[0.5 g FeSO_4_·7H_2_O dissolved in 50 mL H_2_O]+2 mL 10M HCl, with the precipitate, barium sulfate, filtered off to produce a clear solution]) was added and the solutions were vortexed for 2–4 s. After incubation (5 min) at room temperature, absorbance was read at 500 nm against a blank that contained all the reagents except the sample. The calibration curve of Fe(III) concentration versus absorbance (up to ~1.2) was linear in a wide range of Fe(III) concentration (0–40 µg). Limit of detection for PV was as low as 0.2–0.5 mEq kg^–1^. To measure [CD], the oils (0.01–0.03 g) were dissolved in HPLC-grade *n*-hexane (10 mL) and absorbance was read at 234 nm (Pegg, 2001). An extinction coefficient of 29000 L mol^–1^ cm^–1^ was used to calculate the concentration of conjugated dienes (Privett & Blank, 1962).

*2.3. Kinetics of [LOOH] and [CD] accumulations*

Kinetic curves of change in [LOOH] and [CD] (Farhoosh, 2020) were drawn over time *t* (Fig. 1). The linear Eq. [1] was fitted on the initiation phases:

$\text{[}\text{LOOH}\text{]}\text{ (or }\text{[}\text{CD}\text{]}\text{)}=k_{\mathrm{IP}}\left( t \right)+{[LOOH]}_{0} \left( \mathrm{or} [{CD]}_{0} \right) [1]$

where *k*_IP_ (mM h^–1^) is a pseudo-zero order rate constant, and [LOOH]_0_ or [CD]_0_ are the corresponding values at *t* = 0. The sigmoidal Eq. [2] was fitted on the propagation phases:

$$\text{[}\text{LOOH}\text{]}\text{ (or }\text{[}\text{CD}\text{]}\text{) }\text{=}\frac{k_{c}}{\exp\text{(}k_{c}\text{C}-k_{c}\text{t}\text{) + }k_{d}} [2]$$

where *k*_c_ (h^−1^) and *k*_d_ (mM^–1^ h^−1^) are the pseudo-first and -second order composite and decomposition, respectively, rate constants. C is an integration constant. [LOOH]_max_ and [CD]_max_ (mM) were calculated from the ratio *k*_c_/*k*_d_. The x-coordinate of the equation’s turning point gave the time at which [LOOH] reaches the middle point of the propagation phase, *t*_T_ = C – [(ln*k*_d_)/*k*_c_]. The y-coordinate of the point was [LOOH]_T_ = *k*_c_/2*k*_d_. The time required to reach [LOOH]_max_ or [CD]_max_ was *t*_max_ = (2 + *k*_c_C – ln*k*_d_)/*k*_c_. The maximum rate of LOOH and CD accumulations (*r*_max_, mM h^–1^) was obtained by the ratio *k*_c_^2^/4*k*_d_. IP (h) and [LOOH]_IP_ or [CD]_IP_ (mM) were calculated as follows:

$$\text{IP}=\frac{k_{c}\left( 2-k_{c}C+\text{ln}k_{d} \right)-4{[LOOH]}_{o}\left( \mathrm{or} {[CD]}_{o} \right)k_{d}}{4k_{\mathrm{IP}}k_{d}-{k_{c}}^{2}} [3]$$

$${[LOOH]}_{\mathrm{IP}}\left( \mathrm{or} {[CD]}_{\mathrm{IP}} \right)=k_{\mathrm{IP}}\left( \text{IP} \right)+{[LOOH}_{]0}\left( \mathrm{or} {[CD]}_{0} \right) [4]$$

*2.4. Thermodynamics of [LOOH] and [CD] accumulations*

Temperature coefficients (*T*_C_, °C^−1^) were calculated from the linear Eq. [5] (Farhoosh & Hoseini-Yazdi, 2014):

$$\log\left( x \right)=T_{C}\left( \text{T} \right)+log b [5]$$

where *x* can be any of the kinetic parameters stated in the section 2.3, T is temperature (°C), and log *b* is the *y*-intercept. *Q*_10_ number was obtained from Eq. [6] (Toorani, Farhoosh, Golmakani, & Sharif, 2021):

$Q_{10}={10}^{10T_{C}}$	[6]

The semi-logarithmic Arrhenius Eq. [7] was used to calculate activation energies (*E*_a_, kJ mol^–1^) and frequency factors (*A*, the same dimension as *k*):

$$\mathrm{Ln} k=\ln A-\frac{E_{a}}{\mathrm{RT}} [7]$$

where *k* is the rate constants *k*_IP_, *k*_c_, or *k*_d_, R is the molar gas constant (8.3143 J mol^−1^ K^−1^), and T is absolute temperature (K) (Farhoosh & Hoseini-Yazdi, 2014).

The semi-logarithmic Eyring Eq. [8] was used to calculate enthalpies (∆*H*^++^, kJ mol^−1^) and entropies (∆*S*^++^, J mol^−1^ k^−1^) of the activated complexes at standard state:

$$\ln\left( \frac{k}{T} \right)=\ln\left( \frac{k_{B}}{h}\left( C^{^{\circ}} \right)^{1-n} \right)+\frac{{\Delta S}^{++}}{R}-\frac{{\Delta H}^{++}}{\mathrm{RT}} [8]$$

where *k*_B_ is the Boltzmann constant (1.380658×10^−23^ J K^−1^); *h* is Planck’s constant (6.6260755×10^−34^ Js); (*C*°)^1–^*^n^* is the correct dimensionality factor for the reactions that are not of first order kinetics (*C*°: the standard-state concentration of 1 M; *n*: the reaction order (Laidler, 1981).

*2.5. Shelf-life estimation*

Assuming the pseudo-zero order reaction kinetics dominating in the initiation peroxidation phase, shelf-lives (*t*_S1_, h) were estimated by using Eq. [9] (Farhoosh, 2022):

$$t_{S1}=\frac{C_{r}-{[LOOH]}_{0} \left( \mathrm{or} [{CD]}_{0} \right)}{A\exp\left( -\frac{E_{a}}{RT_{a}} \right)} [9]$$

where C_r_ is the cut-off [LOOH] or [CD] value for rejection of the oils, *E*_a_ and *A* are the *k*_IP_-based activation energy and frequency factor, respectively, and T_a_ is ambient temperature (298 K). Moreover, considering the fact that the upper quality limits of lipid systems might be situated shortly after the value of IP (Hamilton, 1994; Moosavi & Farhoosh, 2023), shelf-lives (*t*_S2_, h) were also estimated by using Eq. [10] (Farhoosh, 2022):

$$t_{S2}=C_{a}-\frac{\ln A_{d}-\frac{E_{a,d}}{RT_{a}}}{A_{c}\exp\left( -\frac{E_{a,c}}{RT_{a}} \right)} [10]$$

where *A*_c_, *A*_d_, *E*_a,c_, and *E*_a,d_ are the *k*_c_- and *k*_d_-based frequency factors and activation energies, respectively. C_a_ is the integration constant C at the ambient temperature T_a_ which was simply calculated by extrapolating their values at the accelerated temperatures to 298 K using a linear regression equation (Farhoosh, 2021). To verify the estimated shelf-lives, the values of IP, *t*_T_, and *t*_max_ at 298 K were calculated from Eq. [5] (Frankel, 2012a).

*2.6. Statistical analysis*

All measurements were in triplicate and data were subjected to analysis of variance (ANOVA). ANOVA and regression analyses were performed according to the MStatC and SlideWrite software version 7.0. Significant differences between means were determined by Duncan’s multiple range tests. The values of *p* less than 0.05 were considered statistically significant.

**3. Results and discussion**

*3.1. Kinetics of [LOOH] and [CD] accumulations*

Figure 1 exhibits the kinetic curves of LOOH and CD accumulations over the whole peroxidation range of the oils studied at 45–85°C. The kinetic parameters and rate constants calculated from the linear [1] and sigmoidal [2] equations fitted to the experimental data points in the initiation and propagations phases, respectively, at 45°C with high coefficients of determination (R^2^ > 0.97) are presented in Table 1.

The LOOH- and CD-based IP values as measures of the oxidative reactivity of the peroxyl radicals (LOO^⦁^), resulting from the constituting fatty acids, indicated that the olive, sesame, canola, and corn, respectively, oils were of higher potential to generate more reactive LOO^⦁^. However, the values of the LOOH-based *k*_IP_, which indicate the level of tendency to generate free radicals (e.g., LOO^⦁^, LO^⦁^, and ^⦁^OH) of various reactivity and diversity (Yanishlieva & Marinova, 2003), were significantly higher in the olive, canola, and corn ≈ sesame, respectively, oils. Oxidizability in the initiation phase of lipid peroxidation is essentially affected by the relative amount of unsaturated fatty acids and more importantly the content and potency of naturally occurring and/or added antioxidants in an oil (Farhoosh, 2025) (see the section 2.1). Statistically the same values of IP and *k*_IP_ were observed for LOOH and CD accumulations in each oil, indicating CDOOH to be possibly dominant primary oxidation products over the initiation phase, which were reliably measured by both LOOH and CD assays. This demonstrates the same potency of the two analytical methods in the assessment of oxidizability over the initiation phase of lipid peroxidation.

The values of [LOOH]_IP_, implying the degree of peroxidation occurred at the end of IP, were below 4.0 mM in general. The permissible range of [LOOH] for refined vegetable oils has been promulgated by the Codex Alimentarius (2024) to be ~5.0 mM. The corresponding values of [CD]_IP_ were 10.9 mM, 14.9 mM, and 12.9 mM for the olive, corn/sesame, and canola, respectively, oils, which were partly higher than the suggested cut-off [CD] values of 8.28 mM, 12.4 mM, and 12.0 mM for rejection of the oleic, oleic-linoleic, and linolenic groups of vegetable, respectively, oils (Sarfaraz Khabbaz et al., 2024).

The significantly lowest value of the LOOH-based *k*_c_, which is directly dependent on the *r*_max_ and *k*_d_ values [*k*_c_ = 2(*r*_max_.*k*_d_)^0.5^] (Farhoosh, 2020), was obtained for the olive oil, followed by the sesame, canola, and corn oils. The propagation oxidizability depends mainly on the degree of saturation and mostly the content and inhibitory effect of the endogenous phenolic compounds, due to the almost complete depletion of tocopherols over the initiation phase (Farhoosh, 2025). The comparison between the kinetics of change in LOOH and CD showed higher *k*_c_ values in general for the former. This was due to the bigger *r*_max_ and *k*_d_ values for the LOOH than for the CD formation by the factors of 1.29 – 1.60 and 1.08 – 3.45, respectively. The bigger LOOH-based values of *r*_max_ and *k*_d_ might possibly be owing to the increased oxidative contribution of nCDOOH generated from MUFA peroxidation in the phase, on one hand, and the decreased oxidative contribution of CDOOH, participating in the Diels-Alder reaction, on the other hand (Jaldani et al., 2024). The Diels-Alder reaction is a non-radical cyclization between a conjugated diene and a substituted alkene, forming a substituted cyclohexene unsaturated dimer that is unable to absorb at 234 nm (Frankel, 2012c). The comparative values of the *r*_max_ and *k*_d_ can also explain the variations observed in the kinetic parameters [LOOH]_max_ and [CD]_max_ [= 2(*r*_max_/*k*_d_)^0.5^] (Farhoosh, 2020).

*3.2. Thermodynamics of [LOOH] and [CD] accumulations*

Table 2 shows the semi-logarithmic equations of the LOOH- and CD-based kinetic parameters and rate constants representing the initiation and propagation phases of the oils during peroxidation at 45–85°C. The thermodynamic data calculated on the basis of the rate constants *k*_IP_, *k*_c_, and *k*_d_ is presented in Table 3.

*3.2.1. Temperature acceleration factor*

The temperature coefficient *T*_C_ calculated on time parameters, e.g. IP, *t*_T_, or *t*_max_, may be used to show how lipid systems react against changes in temperature, so that its bigger absolute number reflects higher temperature susceptibility to peroxidation (Farhoosh, 2021). The absolute *T*_C_ values resulting from the temperature dependencies of the parameters IP and *k*_IP_ were generally higher for the corn oil, followed by the sesame, canola, and olive oils (Table 2). However, some statistically different patterns were observed regarding the *T*_C_ values for the LOOH- and CD-based parameters *k*_c_, *k*_d_, and *r*_max_. The temperature dependencies of the initiation and propagation rate constants were in line with the corresponding *Q*_10_ numbers (Table 3), which imply the factors by which the reaction rates increase for every 10°C rise in temperature. The higher temperature susceptibilities naturally results from the chemical compositions containing more polyunsaturated fatty acids as well as the antioxidative components of higher tendency to thermal destructions and/or vaporization, known as carry-through properties (Reische, Lillard, & Eitenmiller, 2008).

Approximately no temperature dependencies were observed for the values of LOOH and CD at the end of the initiation ([LOOH]_IP_ and [CD]_IP_) and propagation ([LOOH]_max_ and [CD]_max_) phases. This was in agreement with the results from our previous study on the thermal peroxidation of canola and sesame oils (Farhoosh, 2021). On this basis, the *y*-intercept (log *b*, Table 2) of the semi-logarithmic Eq. [5] as potential cut-off [LOOH] and [CD] values (C_r_, see the section 2.5) for rejection of the olive, corn, sesame, and canola oils were calculated to be 2.84 mM (5.63 mEq kg^–1^) and 12.6 mM (13.7 µmol g^–1^), 3.68 mM (7.30 mEq kg^–1^) and 15.32 mM (16.7 µmol g^–1^), 2.83 mM (5.61 mEq kg^–1^) and 14.9 mM (16.2 µmol g^–1^), and 3.26 mM (6.46 mEq kg^–1^) and 12.5 mM (13.6 µmol g^–1^), respectively.

*3.2.2. Arrhenius and Eyring equations’ parameters*

The Arrhenius and Eyring equations yielded two valuable sets of equivalent parameters [*E*_a_ ≈ ∆*H*^++^ and *A* = (*k*_B_T/*h*)exp(∆*S*^++^/R)], which fundamentally provide us with a practical understanding on lipid peroxidation as affected by temperature (Farhoosh, 2022). Linear dependencies of the rate constants were observed by rising temperature in the range from 45°C to 85°C. Theoretically, such linearities may imply no change in the mechanism of lipid peroxidation. This was in contrast to Gomez-Alonso et al. (2004) and Frankel (2012b), who postulated that the mechanism of lipid peroxidation is significantly different at temperatures higher than 60°C. In fact, the temperature dependence of the mechanism of the reaction was eliminated by performing lipid peroxidation in a kinetic regime of oxidation (Yanishlieva & Marinova, 2003).

As can be seen in Table 3, the values of *A* and *E*_a_ for the formation of LOOH during the initiation peroxidation phase were significantly higher in the corn oil (1322×10^4^ mM h^−1^ and 57.1 kJ mol^−1^) than in the sesame (248×10^4^ mM h^−1^ and 52.9 kJ mol^−1^), canola (14.7×10^4^ mM h^−1^ and 44.7 kJ mol^−1^), and olive (2.68×10^4^ mM h^−1^ and 37.5 kJ mol^−1^) oils, respectively. *E*_a_ is actually the minimum energy required to transfer reactants from their ground state, i.e. the lowest-energy and most stable state, to a transition state of sufficient energy to generate final reaction products. In the transition state, molecular configuration of the reactants carries distorted chemical bonds that vibrate with higher frequencies, making them more likely to break. On this basis, higher values of *E*_a_ (or ∆*H*^++^) demonstrate higher levels of energy required for the activation of its oxidizing reactants as transient complexes which further dissociate into final products. This, in turn, means greater resistance of the oil against initiation peroxidation. However, bigger values of *A*, which is the ultimate rate constant at extreme conditions theoretically (*E*_a_ = 0 or T→ ∞), indicate faster peroxidation as temperature increases and goes beyond the thermal range experimented (Farhoosh, 2022). The *A* values were in good conformity with the entropies of activation (∆*S*^++^) for the corn (–186 J mol^–1^ K^–1^), sesame (–200 J mol^–1^ K^–1^), canola (–224 J mol^–1^ K^–1^), and olive (–238 J mol^–1^ K^–1^) oils. As for the LOOH formation during the propagation peroxidation phase, the significantly bigger *A* values were observed in the sesame (2940×10^4^ h^−1^), corn (2371×10^4^ h^−1^), canola (876×10^4^ h^−1^), and olive (31.6×10^4^ h^−1^) oils, respectively. However, the corn, sesame, and canola oils were of statistically the same *E*_a_ values (~52 – 57 kJ mol^−1^), which were significantly higher than that of the olive oil (46.0 kJ mol^−1^). The quite smaller *A* value in the olive oil indicated that the oil would be of much lower rate of propagation oxidizability as temperature progressively increased to above 85°C. The preformed activated complexes of significantly lower entropy in the olive oil (∆*S*^++^= –217 J mol^–1^ K^–1^) can explain well such an occurrence thermodynamically. A roughly similar comparative pattern was observed for the decomposition of LOOH molecules.

The values of *E*_a_ (or ∆*H*^++^) for the formation of LOOH were statistically the same as their corresponding equivalents for the formation of CD during the initiation peroxidation phase of the oils (Table 3). However, statistically different values of *A* (or ∆*S*^++^) were obtained for the LOOH and CD formations during the phase. This indicates that the two assays are tracking some different transient complexes carrying oxidizing components of various conformational flexibilities due to the degree of unsaturation (Ghnimi, Budilarto, & Kamal-Eldin, 2017) and, therefore, of various structural orders. In other words, the LOOH and CD assays reflect the effect of temperature on lipid peroxidation differently. Considering the different contribution of unsaturated fatty acids to peroxidation of the oils after the initiation phase (Jooyandeh, Jaldani, & Farhoosh, **2023**), statistically different *k*_c_- and *k*_d_-based thermodynamic factors were also calculated by the two assays.

*3.3. Shelf-life estimation*

In an expected way, the corn oil of significantly lower initiation oxidizability (Table 1) displayed a relatively longer LOOH-based shelf-life (*t*_S1_ = 2210 h) compared with the sesame (1168 h), canola (1052 h), and olive (304 h) oils (Fig. 2). The estimated shelf-life of 2210 h was statistically the same as its *t*_T_ value (1967 h), where the oil could still be consumed with no serious risk of LOOH decomposition into undesirable secondary oxidation products (Moosavi & Farhoosh, 2023; Sarfaraz Khabbaz et al., 2024). However, the highly propagation oxidizability of the corn oil, which in turn made the duration of the phase (*t*_max_ – IP, Fig. 2) quite shorter than IP, caused no significant increase from its *t*_S1_ to *t*_S2_ (2450 h). As for the other oleic-linoleic oil, sesame oil, which was of approximately the same initiation and propagation times, an LOOH-based *t*_S1_ value (1168 h vs. *t*_S2_ = 2941 h) around the corresponding *t*_T_ value (1380 h) was calculated as well. The canola oil, belonging to the linolenic group of vegetable oils, also showed a similar comparative pattern (*t*_S1_ = 1052 h vs. *t*_S2_ = 1941 h, and *t*_T_ = 1194 h). However, the olive oil of the lowest propagation oxidizability (Table 1), which made the duration of the propagation phase exceptionally greater than its initiation phase by a factor of ~3.5 (Fig. 2), demonstrated a *t*_S1_ value (304 h vs. *t*_S2_ = 1145 h) significantly lower than the corresponding *t*_T_ value (721 h). It seems that an average of the *t*_S1_ and *t*_S2_ (725 h) values would provide a more accurate estimation of shelf-life for the olive oil representing the oleic group of vegetable oils.

The CD-based *t*_S1_ values were much lower than the LOOH-based shelf-lives estimated in the corn (1296 h vs. 2210 h), sesame (182 h vs. 1168 h), canola (142 h vs. 1052 h), and olive (457 h vs. 725 h) oils (Fig. 2). However, an average of the CD-based *t*_S1_ and *t*_S2_ values in the corn (2130 h), sesame (1279 h), canola (801 h), and olive (755 h) oils provided roughly the same shelf-lives as those estimated on the basis of LOOH measurements. As discussed kinetically and thermodynamically above, CD represent a less limited range of oxidized fatty acids and this makes us include both *t*_S1_ and *t*_S2_ values in the shelf-life estimations.

**4. Conclusions**

The present study demonstrated that investigating the simultaneous changes in LOOH and CD with temperature may provide many equivalent kinetic and thermodynamic parameters of different quantities. This was due to the fact that LOOH represent exclusively primary oxidation products (nCDOOH + CDOOH) whereas CD stand concomitantly for some primary (CDOOH) and secondary (CDnOOH) oxidation products, which their levels depended dramatically upon the fatty acid composition of the oxidizing oils. The pseudo-zero, -first, and -second order kinetics dominating in the initiation and propagation phases of lipid peroxidation using the kinetic Arrhenius model were adopted to fundamentally estimate model-predicted shelf-lives under the specified experimental conditions. Linear Arrhenius dependencies on the reaction temperatures of 45°C to 85°C were established, which made possible using the accelerated temperatures above 60°C to estimate shelf-lives with probably no change in the mechanism of lipid peroxidation. Both the LOOH- and CD-based temperature-dependent rate constants were practically employed to estimate roughly the same shelf-lives. It should be noticed that the oils in this study were oxidized in a kinetic regime to avoid dependency on oxygen concentration and obtain more reproducible data. In other words, getting more real results requires conducting studies using oil layers thicker than 1 mm, where oxygen diffusivity is the case.

**References**

American Oil Chemists' Society (AOCS). (2017). Official Method Cd 3d-63, Acid Value of Fats and Oils. In *Official Methods and Recommended Practices of the AOCS* (7th ed.). Champaign: AOCS Press.

Capannesi, C., Palchetti, I., Mascini, M., & Parenti, A. (2000). Electrochemical sensor and biosensor for polyphenols detection in olive oils. *Food Chemistry*, 71, 553–562. https://doi.org/[10.1016/S0308-8146(00)00211-9](http://dx.doi.org/10.1016/S0308-8146(00)00211-9)

Codex Alimentarius. Codex Alimentarius Commission. (2024). Standard for named vegetable oils (CXS 210-1999). Codex Alimentarius Commission, Rome.

Farhoosh, R. (2018). Reliable determination of the induction period and critical reverse micelle concentration of lipid hydroperoxides exploiting a model composed of pseudo-first and -second order reaction kinetics. *LWT – Food Science and Technology*, 98, 406–410. https://doi.org/[10.1016/j.lwt.2018.09.003](http://dx.doi.org/10.1016/j.lwt.2018.09.003)

Farhoosh, R. (2020). A reconsidered approach providing kinetic parameters and rate constants to analyze the oxidative stability of bulk lipid systems. *Food Chemistry*, 327, 127088. https://doi.org/10.1016/j.foodchem.2020.127088

Farhoosh, R. (2021). Critical kinetic parameters and rate constants representing lipid peroxidation as affected by temperature. *Food Chemistry*, 340, 128137. https://doi.org/10.1016/j.foodchem.2020.128137

Farhoosh, R. (2022). New insights into the kinetic and thermodynamic evaluations of lipid peroxidation. *Food Chemistry*, 375, 131659. https://doi.org/[10.1016/j.foodchem.2021.131659](https://doi.org/10.1016/j.foodchem.2021.131659)

Farhoosh, R. (2025). Relative contribution of initial quality indicators and chemical composition data to the initiation and propagation oxidizabilities of vegetable oils. *Food Chemistry*, 477, 143545. https://doi.org/10.1016/j.foodchem.2025.143545

Farhoosh, R., & Hoseini-Yazdi, S.Z. (2014). Evolution of oxidative values during kinetic studies on olive oil oxidation in the Rancimat test. *Journal of the American Oil Chemists’ Society*, 91, 281–293. https://doi.org/10.1007/s11746-013-2368-z

Farhoosh, R., Niazmand, R., Rezaei, M., & Sarabi, M. (2008). Kinetic parameter determination of vegetable oil oxidation under Rancimat test conditions. ***European Journal of Lipid Science and Technology*,** 110, 587–592. https://doi.org/10.1002/ejlt.200800004

Frankel, E.N. (2012a). Methods to determine extent of oxidation. In E.N. Frankel (Ed.), *Lipid Oxidation* (pp. 99–127). Cambridge: Woodhead Publishing Limited.

Frankel, E.N. (2012b). Stability methods. In E.N. Frankel (Ed.), *Lipid Oxidation* (pp. 165–186). Cambridge: Woodhead Publishing Limited.

Frankel, E.N. (2012c). Frying fats. In E.N. Frankel (Ed.), *Lipid Oxidation* (pp. 355–390). Cambridge: Woodhead Publishing Limited.

Ghnimi, S., Budilarto, E., & Kamal-Eldin, A. (2017). The new paradigm for lipid oxidation and insights to microencapsulation of omega-3 fatty acids. *Comprehensive Reviews in Food Science and Food Safety*, 16, 1206–1218. <https://doi.org/10.1111/1541-4337.12300>

Gomez-Alonso, S., Salvador, M.D., & Fregapane, G. (2004). Evolution of the oxidation process in olive oil triacylglycerol under accelerated storage conditions (40–60°C). *Journal of the American Oil Chemists’ Society*, 81, 177–184. https://doi.org/10.1007/s11746-004-0878-7

Hamilton, R.J. (1994). The chemistry of rancidity in foods. In J.C. Allen, & R.J. Hamilton (Eds.), *Rancidity in Foods* (3rd edition, pp. 1–21). London: Blackie Academic and Professional.

Jaldani, S., Sarfaraz Khabbaz, E., Jooyandeh, M., & Farhoosh, R. (2024). Kinetics of simultaneous change in the concentration of total lipid hydroperoxides and total conjugated dienes during peroxidation of canola, sunflower, and olive oils. *Food Chemistry*, 435, 137605. https://doi.org/10.1016/j.foodchem.2023.137605

Jooyandeh, M., Jaldani, S., & Farhoosh, R. **(2023)**. Stepwise peroxidation of canola and olive oils: A kinetic study. Journal of the American Oil Chemists' Society, 100, 975–983. https://doi.org/10.1002/aocs.12756

Kamal-Eldin, A., & Yanishlieva, N. (2005). Kinetic analysis of lipid oxidation data. In A. Kamal-Eldin, & J. Pokorny (Eds.), *Analysis of Lipid Oxidation* (pp. 234–263). Champaign, Illinois: AOCS Press.

Laidler, K.J. (1981). Symbolism and terminology in chemical kinetics. *Pure and Applied Chemistry*, 53, 753–771.

Moosavi, S.M.R., & Farhoosh, R. (2023). A New insight into the evaluation of used frying oils based on the kinetics of chemical changes during the process. *Foods*, 12, 316. https://doi.org/10.3390/foods12020316

Pegg, R.B. (2001). Measurement of primary lipid oxidation products. *Current Protocols in Food Analytical Chemistry*. John Wiley & Sons, Inc.

Privett, O.S., & Blank, M.L. (1962). The initial stages of autoxidation. *Journal of the American Oil Chemists’ Society*, 39, 465–469. <https://doi.org/10.1007/BF02637226>

Reische, D.W., Lillard, D.A., & Eitenmiller, R.R. (2008). In C.C. Akoh, & D.B. Min (Eds.), *Food Lipids: Chemistry, Nutrition, and Biotechnology* (pp. 409–433). Boca Raton: CRC Press.

Sarfaraz Khabbaz, E., Jooyandeh, M., Jaldani, S., & Farhoosh, R. (2024). Cut-off conjugated diene values for rejection of vegetable oils. *LWT – Food Science and Technology*, 192, 115712. https://doi.org/10.1016/j.lwt.2023.115712

Schaich, K.M. (2005). Lipid oxidation in fats and oils: Theoretical aspects. In F. Shahidi (Ed.), *Bailey’s Industrial Fats and Oils* (6th ed., pp. 2681–2767). New York: John Wiley.

Shantha, N.C., & Decker, E.A. (1994). Rapid, sensitive, iron-based spectrophotometric methods for determination of peroxide values of food lipids. *Journal of AOAC International*, 77, 421–424. <https://doi.org/10.1093/jaoac/77.2.421>

Toorani, M.R., Farhoosh, R., Golmakani, M.T., & Sharif, A. (2021). Thermal antioxidative kinetics of sesamol in triacylglycerols and fatty acid methyl esters of sesame, olive, and canola oils. *Journal of the American Oil Chemists’ Society*, 98, 871–880. https://doi.org/10.1002/aocs.12520

Wong, M.L., Timms, R. E., & Goh, E. M. (1988). Colorimetric determination of total tocopherols in palm oil, olein and stearin. *Journal of the American Oil Chemists’ Society*, 65, 258–261. https://doi.org/10.1007/BF02636412

Yanishlieva, N.V., & Marinova, E.M. (2003). Kinetic evaluation of the antioxidant activity in lipid oxidation. In A. Kamal-Eldin (Ed.). *Lipid Oxidation Pathways* (pp. 85–110). Champaign: AOCS Press.

**Figure 1.** Kinetic curves of the accumulation of lipid hydroperoxides (LOOH) and conjugated dienes (CD) over peroxidation of the oils at 45–85°C.

**Figure 2.** The LOOH (lipid hydroperoxides)- and CD (conjugated dienes)-based shelf-lives (*t*_S1_ and *t*_S2_) estimated for the vegetable oils at 25°C. IP: induction period; *t*_T_: the time at which [LOOH] or [CD] reaches the middle point of the propagation phase; *t*_max_: the time required to reach [LOOH]_max_ or [CD]_max_. Means ± SD (standard deviation) for any of the oils with the same lowercase letters are not signiﬁcantly different at *p* < 0.05.

**Table 1.** Kinetics of change in the concentration of lipid hydroperoxides ([LOOH], mM) and conjugated dienes ([CD], mM) over the initiation and propagation peroxidation phases of the oils at 45°C. ^a^

| Kinetic parameter | Olive oil | |  | Corn oil | |  | Sesame oil | |  | Canola oil | |
| --- | --- | --- | --- | --- | --- | --- | --- | --- | --- | --- | --- |
|  | LOOH | CD |  | LOOH | CD |  | LOOH | CD |  | LOOH | CD |
| IP ^b^  *k*_IP_ ^c^  [LOOH]_IP_/[CD]_IP_ ^d^  *k*_c_ ^e^  *k*_d_ (×10^–4^) ^f^  *r*_max_ ^g^  [LOOH]_max_/[CD]_max_ ^h^ | 146 ± 2 d  0.0174 ± 0.0009 a  3.26 ± 0.17 ef  0.0090 ± 0.0002 d  3.03 ± 0.17 c  0.0664 ± 0.0016 d  29.6 ± 1.2 cd | 125 ± 14 d  0.0190 ± 0.0009 a  10.9 ± 0.2 c  0.0076 ± 0.0004 e  2.80 ± 0.14 c  0.0516 ± 0.0024 e  27.1 ± 0.4 e |  | 520 ± 13 a  0.0059 ± 0.0001 de  3.79 ± 0.06 d  0.0227 ± 0.0009 a  6.15 ± 0.21 a  0.2084 ± 0.0100 a  36.8 ± 0.3 b | 512 ± 9 a  0.0053 ± 0.0002 e  14.9 ± 0.2 a  0.0119 ± 0.0008 c  2.68 ± 0.21 c  0.1306 ± 0.0083 b  44.2 ± 1.6 a |  | 296 ± 9 c  0.0055 ± 0.0003 de  2.90 ± 0.09 f  0.0126 ± 0.0004 c  4.04 ± 0.21 b  0.0988 ± 0.0012 c  31.3 ± 0.7 c | 277 ± 6 c  0.0061 ± 0.0003 cd  14.9 ± 0.2 a  0.0055 ± 0.0007 f  1.17 ± 0.28 d  0.0639 ± 0.0014 d  47.2 ± 5.0 a |  | 354 ± 6 b  0.0072 ± 0.0001 b  3.49 ± 0.10 e  0.0188 ± 0.0013 b  6.15 ± 0.54 a  0.1430 ± 0.0081 b  30.5 ± 1.0 cd | 368 ± 15 b  0.0071 ± 0.0007 bc  12.9 ± 0.5 b  0.0127 ± 0.0005 c  4.35 ± 0.10 b  0.0934 ± 0.0045 c  29.4 ± 0.4 d |

^a^ Means ± SD (standard deviation) within a row with the same lowercase letters are not signiﬁcantly different at *p* < 0.05. ^b^ Induction period (h); ^c^ Pseudo-zero order rate constant of initiation phase (mM h^–1^); ^d^ LOOH or CD concentration (mM) at IP; ^e^ Pseudo-first order composite rate constant (h^–1^); ^f^ Pseudo-second order decomposition rate constant (mM^–1^ h^–1^); ^g^ Maximum rate of LOOH or CD accumulation (mM h^–1^); ^h^ Maximum concentration of LOOH or CD (mM).

**Table 2.** Temperature coefficient (*T*_C_, °C^–1^) for the kinetic parameters and rate constants representing the initiation and propagation phases of the accumulation of lipid hydroperoxides (LOOH) and conjugated dienes (CD) over peroxidation of the oils at T = 45–85°C. ^a^

|  | log (y) = *T*_C_ (T) + log *b* | | | | | | | | | | | | |  |
| --- | --- | --- | --- | --- | --- | --- | --- | --- | --- | --- | --- | --- | --- | --- |
| Kinetic  parameter (y) | Olive oil | |  | Corn oil | |  |  | Sesame oil | |  |  | Canola oil | |  |
|  | *T*_C_ (×10^–2^) | log *b* |  | *T*_C_ (×10^–2^) | log *b* | |  | *T*_C_ (×10^–2^) | log *b* | |  | *T*_C_ (×10^–2^) | log *b* | |
| LOOH  IP ^b^  *k*_IP_ ^c^  [LOOH]_IP_ ^d^  *t*_T_ ^e^  *t*_max_ ^f^  *k*_c_ ^g^  *k*_d_ ^h^  *r*_max_ ^i^  [LOOH]_max_ ^j^  CD  IP ^b^  *k*_IP_ ^c^  [CD]_IP_ ^d^  *t*_T_ ^e^  *t*_max_ ^f^  *k*_c_ ^g^  *k*_d_ ^h^  *r*_max_ ^i^  [CD]_max_ ^j^ | –1.79 ± 0.16 cA  1.71 ± 0.13 cA  –  –1.93 ± 0.11 bA  –2.00 ± 0.11 bA  2.11 ± 0.12 bA  2.03 ± 0.14 bA  2.20 ± 0.15 bA  –  –2.02 ± 0.11 cA  1.56 ± 0.07 cA  –  –1.76 ± 0.14 cA  –1.82 ± 0.10 cA  1.87 ± 0.07 bA  1.81 ± 0.19 abA  1.94 ± 0.06 bA  – | 2.91 ± 0.02 dB  –2.48 ± 0.01 dB  0.45 ± 0.04 bB  3.34 ± 0.01 dA  3.61 ± 0.01 dA  –2.99 ± 0.01 bA  –4.40 ± 0.01 bA  –2.18 ± 0.02 aA  1.47 ± 0.03 aA  2.98 ± 0.01 dA  –2.40 ± 0.01 dA  1.00 ± 0.03 cA  3.10 ± 0.02 dB  3.50 ± 0.01 dB  –2.99 ± 0.01 cA  –4.44 ± 0.02 cA  –2.14 ± 0.01 cA  1.50 ± 0.04 bA |  | –2.60 ± 0.09 aA  2.62 ± 0.06 aA  –  –2.60 ± 0.07 aA  –2.60 ± 0.06 aA  2.54 ± 0.11 aA  2.72 ± 0.08 aA  2.36 ± 0.24 abA  –  –2.52 ± 0.05 aA  2.74 ± 0.09 aA  –  –2.46 ± 0.04 aA  –2.47 ± 0.07 aA  2.51 ± 0.26 aA  2.17 ± 0.17 aB  2.85 ± 0.36 aA  – | 3.89 ± 0.01 aA  –3.42 ± 0.01 aA  0.57 ± 0.02 aB  3.95 ± 0.01 aA  4.01 ± 0.01 aA  –2.82 ± 0.01 cA  –4.43 ± 0.01 bA  –1.81 ± 0.02 dA  1.50 ± 0.06 aB  3.85 ± 0.01 aB  –3.53 ± 0.01 aB  1.19 ± 0.01 aA  3.87 ± 0.00 aB  3.99 ± 0.01 aA  –3.06 ± 0.03 bB  –4.55 ± 0.02 bB  –2.18 ± 0.04 bB  1.71 ± 0.06 aA | |  | –2.38 ± 0.14 abA  2.43 ± 0.11 aA  –  –2.45 ± 0.13 aA  –2.49 ± 0.12 aA  2.62 ± 0.13 aA  2.71 ± 0.19 aA  2.53 ± 0.07 aB  –  –2.24 ± 0.13 bcA  2.24 ± 0.09 bA  –  –2.19 ± 0.12 bA  –2.31 ± 0.09 abA  2.45 ± 0.07 aA  2.16 ± 0.14 aB  2.74 ± 0.04 aA  – | 3.58 ± 0.02 bA  –3.37 ± 0.01 bB  0.45 ± 0.02 bB  3.75 ± 0.01 bA  3.90 ± 0.01 bA  –3.07 ± 0.01 aA  –4.60 ± 0.02 aA  –2.14 ± 0.01 bA  1.47 ± 0.03 aB  3.48 ± 0.01 cB  –3.26 ± 0.01 bA  1.17 ± 0.01 aA  3.61 ± 0.01 cB  3.93 ± 0.01 bA  –3.35 ± 0.01 aB  –4.88 ± 0.01 aB  –2.42 ± 0.01 aB  1.72 ± 0.05 aA | |  | –2.11 ± 0.06 bA  2.06 ± 0.10 bA  –  –2.15 ± 0.05 bA  –2.20 ± 0.04 bA  2.41 ± 0.08 aA  2.44 ± 0.15 aA  2.40 ± 0.13 abA  –  –2.51 ± 0.09 abB  1.98 ± 0.14 bA  –  –2.34 ± 0.09 abA  –2.15 ± 0.06 bA  1.78 ± 0.02 bB  1.61 ± 0.07 bB  1.94 ± 0.09 bB  – | 3.51 ± 0.01 cB  –3.09 ± 0.01 cA  0.51 ± 0.03 abB  3.62 ± 0.01 cB  3.72 ± 0.01 cA  –2.78 ± 0.01 dB  –4.27 ± 0.02 cB  –1.90 ± 0.01 cB  1.47 ± 0.04 aA  3.73 ± 0.01 bA  –3.08 ± 0.02 cA  1.10 ± 0.01 bA  3.68 ± 0.01 bA  3.73 ± 0.01 cA  –2.70 ± 0.00 dA  –4.11 ± 0.01 dA  –1.87 ± 0.01 dA  1.53 ± 0.04 bA | |

^a^ Means ± SD (standard deviation) for the same linear equation’s parameters within a row with the same lowercase letters are not signiﬁcantly different at *p* < 0.05. Means ± SD for the same kinetic parameters within a column with the same uppercase letters are not signiﬁcantly different at *p* < 0.05. Coefficients of determination (R^2^) for the fitted linear functions were higher than 0.95. ^b^ Induction period (h); ^c^ Pseudo-zero order rate constant of initiation phase (mM h^–1^); ^d^ LOOH or CD concentration (mM) at IP; ^e^ The time at which [LOOH] or [CD] reaches the middle point of the propagation phase; ^f^ The time required to reach [LOOH]_max_ or [CD]_max_; ^g^ Pseudo-first order composite rate constant (h^–1^); ^h^ Pseudo-second order decomposition rate constant (mM^–1^ h^–1^); ^i^ Maximum rate of LOOH or CD accumulation (mM h^–1^); ^j^ Maximum concentration of LOOH or CD (mM).

**Table 3.** Thermodynamic data from the rate constants (*k*_IP_, *k*_c_, and *k*_d_) representing the different accumulation phases of lipid hydroperoxides (LOOH) and conjugated dienes (CD) over peroxidation of the oils at 45–85°C. ^a^

|  | Olive oil | |  | Corn oil | |  | Sesame oil | |  | Canola oil | |
| --- | --- | --- | --- | --- | --- | --- | --- | --- | --- | --- | --- |
|  | LOOH | CD |  | LOOH | CD |  | LOOH | CD |  | LOOH | CD |
| Q_10_ ^b^  *k*_IP_ ^g^  *k*_c_ ^h^  *k*_d_ ^i^  *A* (×10^4^)^c^  *k*_IP_ ^g^  *k*_c_ ^h^  *k*_d_ ^i^  *E*_a_ ^d^  *k*_IP_ ^g^  *k*_c_ ^h^  *k*_d_ ^i^  ∆*H*^++^ ^e^  *k*_IP_ ^g^  *k*_c_ ^h^  *k*_d_ ^i^  ∆*S*^++^ ^f^  *k*_IP_ ^g^  *k*_c_ ^h^  *k*_d_ ^i^ | 1.48 ± 0.11 cd  1.63 ± 0.09 ab  1.60 ± 0.11 bc  2.68 ± 0.00 g  31.6 ± 0.1 f  0.60 ± 0.00 f  37.5 ± 2.1 de  46.0 ± 2.9 bc  44.3 ± 3.2 d  34.7 ± 2.2 de  43.1 ± 2.8 bc  41.5 ± 3.2 d  –238 ± 2 b  –217 ± 1 b  –250 ± 4 c | 1.43 ± 0.07 d  1.54 ± 0.06 b  1.52 ± 0.16 bc  0.77 ± 0.00 h  3.47 ± 0.01 g  0.06 ± 0.00 g  34.1 ± 1.1 e  40.8 ± 2.1 cd  39.1 ± 4.7 de  31.3 ± 1.1 e  38.0 ± 2.0 cd  36.3 ± 4.6 de  –248 ± 1 a  –235 ± 1 a  –269 ± 3 b |  | 1.83 ± 0.04 ab  1.79 ± 0.07 a  1.87 ± 0.05 a  1322 ± 2 b  2371 ± 4 b  322 ± 1 a  57.1 ± 1.6 a  55.2 ± 2.8 a  59.3 ± 2.3 a  54.3 ± 1.6 a  52.4 ± 2.8 a  56.5 ± 2.3 a  –186 ± 1 g  –181 ± 1 e  –198 ± 1 e | 1.88 ± 0.06 a  1.78 ± 0.19 ab  1.65 ± 0.13 abc  3067 ± 4 a  1074 ± 4 c  1.57 ± 0.01 d  59.7 ± 2.7 a  54.7 ± 5.5 ab  47.4 ± 3.5 bcd  56.9 ± 2.3 a  51.9 ± 5.5 ab  44.6 ± 3.5 bcd  –179 ± 1 h  –189 ± 1 d  –242 ± 3 c |  | 1.75 ± 0.08 abc  1.83 ± 0.09 a  1.87 ± 0.13 ab  248 ± 1 c  2940 ± 6 a  198 ± 1 b  52.9 ± 2.5 ab  57.1 ± 3.2 a  59.0 ± 4.5 ab  50.1 ± 2.5 ab  54.3 ± 3.2 a  56.2 ± 4.5 ab  –200 ± 1 f  –179 ± 1 e  –202 ± 2 e | 1.68 ± 0.07 bc  1.76 ± 0.05 a  1.65 ± 0.11 abc  54.6 ± 0.1 d  326 ± 1 e  0.65 ± 0.00 e  48.7 ± 2.7 bc  53.4 ± 1.7 a  47.1 ± 3.1 cd  45.9 ± 2.7 bc  50.6 ± 1.7 a  44.3 ± 3.1 cd  –213 ± 1 e  –198 ± 1 c  –249 ± 4 c |  | 1.61 ± 0.08 cd  1.74 ± 0.06 a  1.75 ± 0.11 ab  14.7 ± 0.0 e  876 ± 1 d  37.3 ± 0.1 c  44.7 ± 2.6 c  52.7 ± 1.1 a  53.3 ± 2.5 abc  41.9 ± 2.5 c  49.9 ± 1.1 a  50.5 ± 2.5 abc  –224 ± 1 d  –189 ± 0 d  –216 ± 1 d | 1.58 ± 0.12 cd  1.51 ± 0.02 b  1.45 ± 0.06 c  7.15 ± 0.02 f  2.93 ± 0.00 h  0.02 ± 0.00 h  42.9 ± 2.6 cd  38.8 ± 0.7 d  35.0 ± 2.0 e  40.1 ± 2.6 cd  36.0 ± 0.7 d  32.2 ± 2.0 e  –229 ± 2 c  –237 ± 1 a  –277 ± 1 a |

^a^ Means ± SD within a row with the same lowercase letters are not signiﬁcantly different at *p* < 0.05. ^b^ Temperature acceleration factor; ^c^ Frequency factor with the same dimension as the corresponding rate constant; ^d^ Activation energy (kJ mol^–1^); ^e^ Enthalpy of activation (kJ mol^–1^); ^f^ Entropy of activation (J mol^–1^ K^–1^); ^g^ Pseudo-zero order rate constant of initiation phase (mM h^–1^); ^h^ Pseudo-first order composite rate constant (h^–1^); ^i^ Pseudo-second order decomposition rate constant (mM^–1^ h^–1^).
